# Hypoxia-inducible factor prolyl hydroxylase domain inhibitor may maintain hemoglobin synthesis at lower serum ferritin and transferrin saturation levels than darbepoetin alfa

**DOI:** 10.1371/journal.pone.0252439

**Published:** 2021-06-18

**Authors:** Chie Ogawa, Ken Tsuchiya, Naohisa Tomosugi, Kunimi Maeda

**Affiliations:** 1 Maeda Institute of Renal Research, Kawasaki, Kanagawa, Japan; 2 Biomarker Society, Inc., Kawasaki, Kanagawa, Japan; 3 Department of Blood Purification, Tokyo Women’s Medical University, Tokyo, Japan; 4 Division of Systems Bioscience for Drug Discovery Project Research Center, Medical Research Institute, Kanazawa Medical University, Ishikawa, Japan; National Institute of Child Health and Human Development, UNITED STATES

## Abstract

**Background:**

Hypoxia-inducible factor (HIF) prolyl hydroxylase domain inhibitors, which have recently become clinically available for treating renal anemia, are attracting attention for their novel mechanisms of action.

**Methods:**

Relationships of reticulocyte hemoglobin content (CHr), which reflects recent Hb synthesis, with serum ferritin (s-ft) and transferrin saturation (TSAT) were examined in 30 patients on hemodialysis after switching from darbepoetin alfa (DA) to roxadustat (Rox). Iron deficiency was defined as CHr < 32.0 pg. Cutoff values of s-ft and TSAT were determined using receiver operating characteristic curves for the endpoint CHr ≥ 32.0 pg. Logistic analysis was performed with the reference group having s-ft or TSAT below the corresponding cutoff value (low vs high).

**Results:**

With the endpoint CHr ≥ 32.0 pg on Day 0, cutoff values for s-ft and TSAT were respectively 49.7 ng/mL and 21.6% on Day 0 and 35.5 ng/mL and 16.2% on Day 28. With the endpoint CHr ≥ 32.0 pg on Day 28, cutoff values for s-ft and TSAT on Day 0 were 81.6 ng/mL and 23.9%, respectively. According to multivariable logistic analysis, the odds ratios of CHr ≥ 32.0 pg on Day 0 were significantly higher for high TSAT on Day 0 [34.7 (95% CI 2.42–131.0), p<0.003] and Day 28 [24.8 (95% CI 2.75–224.0), p = 0.004]. There were no significant differences by s-ft. Odd ratios of CHr ≥ 32.0 pg on Day 28 were also significantly higher for high s-ft on Day 0 [16.0 (95% CI 1.57–163.0), p = 0.019] and high TSAT on Day 0 [13.5 (95% CI 1.24–147.0), p<0.033].

**Conclusions:**

Our results suggest Hb synthesis was maintained with lower TSAT and s-ft during Rox therapy compared with DA therapy. To avoid iron deficiency during the 4 weeks after switching DA to Rox, ideal s-ft and TSAT levels before the switch are 81.6 ng/mL and 23.9%, respectively.

## Introduction

Renal anemia is mainly attributed to impairment of erythropoietin production in the kidneys. Over the past 30 years since human recombinant erythropoietin became available for use in patients on dialysis in 1990, erythropoietin-stimulating agents (ESAs), including long-acting ESAs, have been a mainstay of treatment for renal anemia. However, approximately 5–10% of patients show resistance to ESAs, mainly due to impaired iron metabolism associated with malnutrition and inflammation [1, 2].

Hypoxia inducible factor (HIF) prolyl hydroxylase domain (PHD) inhibitors, which are agents with novel mechanisms of action, have recently become available for patients on dialysis and are expected to be effective for ESA-resistant cases. Discovered in 1992, HIF is a transcription factor involved in EPO production induced by hypoxia [3]. At normal oxygen levels, HIF undergoes hydroxylation by PHD and ubiquitination by the von Hippel–Lindau protein, followed by rapid proteasome-dependent degradation. Oxygen molecules, iron, and oxoglutaric acid are essential for activation of PHD. Thus, under hypoxic conditions, PHD activity is reduced and HIF is stabilized to increase expression of genes (e.g., the EPO gene) that are necessary under such conditions. By this mechanism, HIF-PHD inhibitors enhance endogenous EPO production. In addition, HIF2a has been reported to be involved in the regulation of EPO in the liver [4], so it is also possible that there is a mechanism that works well in patients on long-term dialysis who have significant degradation of the renal parenchyma or who have undergone nephrectomy.

One of the major characteristics of HIF is that it improves iron metabolism. Iron is a major component of Hb, but the iron content in blood is only enough to sustain a few hours of hematopoiesis. Thus, rapid iron supply is essential for effective hematopoiesis. Hepcidin is the key regulator of iron supply via binding to ferroportin. Ferroportin is responsible for iron transport from cells to the circulation, and the degradation of ferroportin upon hepcidin binding results in suppression of iron supply from cells to the blood [5]. In addition to suppression of hepcidin production [6–8], HIF induces production of ferroportin [9], thus facilitating iron supply to the circulation. Also, it has been reported that HIF induces various proteins involved in iron metabolism, such as those involved in iron transport, cellular iron uptake, and intestinal iron absorption [10–13].

Although iron utilization and hematopoiesis are impaired in the inflammatory state because iron and inflammatory signals increase the expression of hepcidin [14], phase 3 clinical studies have demonstrated the efficacy of HIF-PHD inhibitors in patients with high C-reactive protein levels [15, 16]. These findings suggest the possibility of using HIF-PHD inhibitors to control iron metabolism, which was difficult to achieve with ESAs.

Hematopoiesis through induction of endogenous EPO production by oral administration of HIF-PHD inhibitors (3 days per week or daily) and that through temporary increases in EPO concentration by intravenous injection of ESAs may be different; moreover, iron requirements may differ between them. Indeed, we previously showed that hematopoiesis and iron utilization were increased immediately after the switch from the long-acting ESA darbepoetin α (DA), to the HIF-PHD inhibitor roxadustat (Rox), suggesting the possibility that the optimal iron conditions are different when using DA and when using Rox [17].

This study examined the iron requirements to avoid iron deficiency and induce effective hematopoiesis before and after a medication switch from DA to Rox.

## Materials and methods

### Patients

In this study, we reanalyzed using a portion of the data from our previous study [17]. The participants in this study were 32 patients receiving outpatient hemodialysis (HD) therapy at our hospital who were being treated with DA for renal anemia and switched to Rox. Patients with fundal hemorrhage or malignancy were excluded. All patients were followed for 4 weeks and received 3 sessions of HD therapy per week, each lasting for 4–5 h.

All patients gave written informed consent to participate in this study. The study protocol was approved by the Ethics Committee of Biomarker Society, Inc., which consists of 7 members, including outside experts (approval number 2020–01), and the study was conducted in accordance with the Declaration of Helsinki.

### Methods

Treatment for anemia was provided with a target Hb level of 10–12 g/dL according to the guidelines of the Japanese Society of Dialysis Therapy. The dose of DA was adjusted accordingly. Rox administration was continued at the initial dose according to the package insert, and no iron supplement or iron-containing phosphate adsorbent was used during the observation period.

Rox administration was started 1 week after the last dose of DA. Rox was administered 3 times weekly at each dialysis session. The administered dose was 70 mg in patients who had received DA at doses of < 20 μg/week and 100 mg in those who had received DA at doses of ≥ 20 μg/week, following the directions on the package insert.

Blood samples were collected at the start of HD on the first day of Rox administration (Day 0) and Day 28 to measure mature erythrocytes, reticulocyte indices (Ret count, CHr), iron-related factors, hepcidin levels, and EPO concentration. TSAT was calculated as serum iron concentration divided by total iron-binding capacity multiplied by 100. Erythrocyte lineage cells were counted using an ADVIA2120 hematology analyzer (Siemens Healthcare Diagnostics, Tarrytown, NY), EPO concentration was measured by chemiluminescent enzyme immunoassay, and hepcidin levels were quantitatively measured by liquid chromatography coupled with tandem mass spectrometry [18].

### Statistical analysis

Data are presented as the mean ± SD or median (interquartile range). The paired t-test was used to compare normally distributed continuous variables between Day 0 and 28, and the Wilcoxon signed rank test was used to compare non-normally distributed continuous variables between these days. The diagnostic criterion for iron deficiency was CHr < 32.0 pg. The optimal cutoff values of ferritin and TSAT for CHr ≥ 32.0 pg were determined by receiver operating characteristic (ROC) analysis with the Youden index. We used univariable and multivariable logistic regression models to evaluate the impact of ferritin and TSAT on CHr ≥32.0 pg. Differences in the slopes of regression models were tested using a generalized linear regression model with an interaction term, and multiple regression analyses examined factors related to the change in Ret count. All analyses were performed using SAS ver. 9.3 software (SAS Institute, Cary, NC). Two-tailed P-values less than 0.05 were considered to indicate statistical significance.

## Results

### Patients

Two patients were excluded during the observation period, one due to stroke and the other due to gastrointestinal hemorrhage. Thus, the remaining 30 patients (17 men, 13 women) were included in the analysis. Mean age was 71.3±12.4 years. Median duration of HD was 7.2 (2.8–16.6) years. The most common underlying disease was diabetic nephropathy, which was present in 14 patients (46.7%). Mean dose of DA before the switch was 18.3±14.7 μg/week. The Rox dose was 70 and 100 mg in 19 and 11 patients, respectively (Table 1).

**Table 1:** Patient characteristics.

Variables	All
N	30
Age (years)	71.3 ± 12.5
Sex	
Men	17
Women	13
Duration of dialysis (years) †	7.2 (2.8–16.6)
Primary diagnosis	
Chronic glomerulonephritis	7
Diabetes nephropathy	14
Renal sclerosis	8
Other	1
Kt/V	1.50 ± 0.20
Albumin (g/dL)	3.5 ± 0.3
C-reactive protein (mg/dL) †	0.12 (0.07–0.60)
Darbepoetin α (μg/week)	18.3 ± 14.7
Roxdustat 70mg	19
100mg	11

Values are shown as the number, mean ± standard deviation, or median (interquartile range).

### Comparison of erythrocytes, reticulocytes, iron-related factors, and erythropoietin on Day 0 and Day 28

Mean red blood cell count (RBC), hemoglobin (Hb) level, reticulocyte (Ret) count, and total iron-binding capacity (TIBC) were significantly increased on Day 28 compared with Day 0. Mean corpuscular volume (MCV), CHr level, serum ferritin level (s-ft), transferrin saturation (TSAT), serum iron level (s-Fe), transferrin saturation (TSAT), and hepcidin level were significantly decreased on Day 28 compared with Day 0 (Table 2).

**Table 2:** Comparison of erythrocytes, reticulocytes, iron-related factors, and erythropoietin.

Variables	Day 0	Day 28	P-value
Red blood cells (×10^4^/μL)	350.2±26.9	371.7±38.9	<0.001
Hemoglobin (g/dL)	10.6±0.7	11.1±0.8	<0.001
MCV (fL)	98.1±5.4	97.1±5.3	0.018
MCH (pg)	30.4±1.7	30.6±1.3	0.467
Reticulocytes (×10^3^/μL)	41.4±15.1	58.2±19.6	<0.001
CHr (pg)	32.4±1.7	31.3±2.5	0.004
Serum ferritin (ng/mL)	48.2 (31.4–97.8)	33.4 (19.3–53.8)	<0.001
Iron (μg/dL)	63.5±22.4	49.4±18.7	0.005
TIBC (μg/dL)	254.2±32.3	330.3±46.1	<0.001
Transferrin saturation (%)	25.2±8.4	15.4±6.5	<0.001
Hepcidin (ng/mL)	26.8 (11.6–58.4)	1.5 (1.0–12.7)	<0.001
erythropoietin (mIU/mL)	11.6 (6.9–17.6)	6.7 (3.1–11.7)	0.052

Values are shown as the mean ± standard deviation or median (interquartile range).

Abbreviations: MCV, mean corpuscular volume; MCH, mean corpuscular hemoglobin; CHr, reticulocyte hemoglobin content; TIBC, total iron-binding capacity.

### CHr levels and iron status on Day 0

According to ROC analysis with CHr ≥ 32.0 pg on Day 0 set as the endpoint, the cutoff value for s-ft was ≥ 49.7 ng/mL (sensitivity 58.3%, specificity 61.1%, area under the curve [AUC] 0.51, 95% confidence interval [CI] 0.30–0.73), whereas the cutoff values for TSAT and s-Fe were respectively ≥ 21.6% (sensitivity 66.7%, specificity 88.9%, AUC 0.83, 95% CI 0.66–1.00) and 57.0 μg/dL (sensitivity 66.7%, specificity 94.4%, AUC 0.79, 95% CI 0.60–0.97) on Day 0.

**Fig 1:**
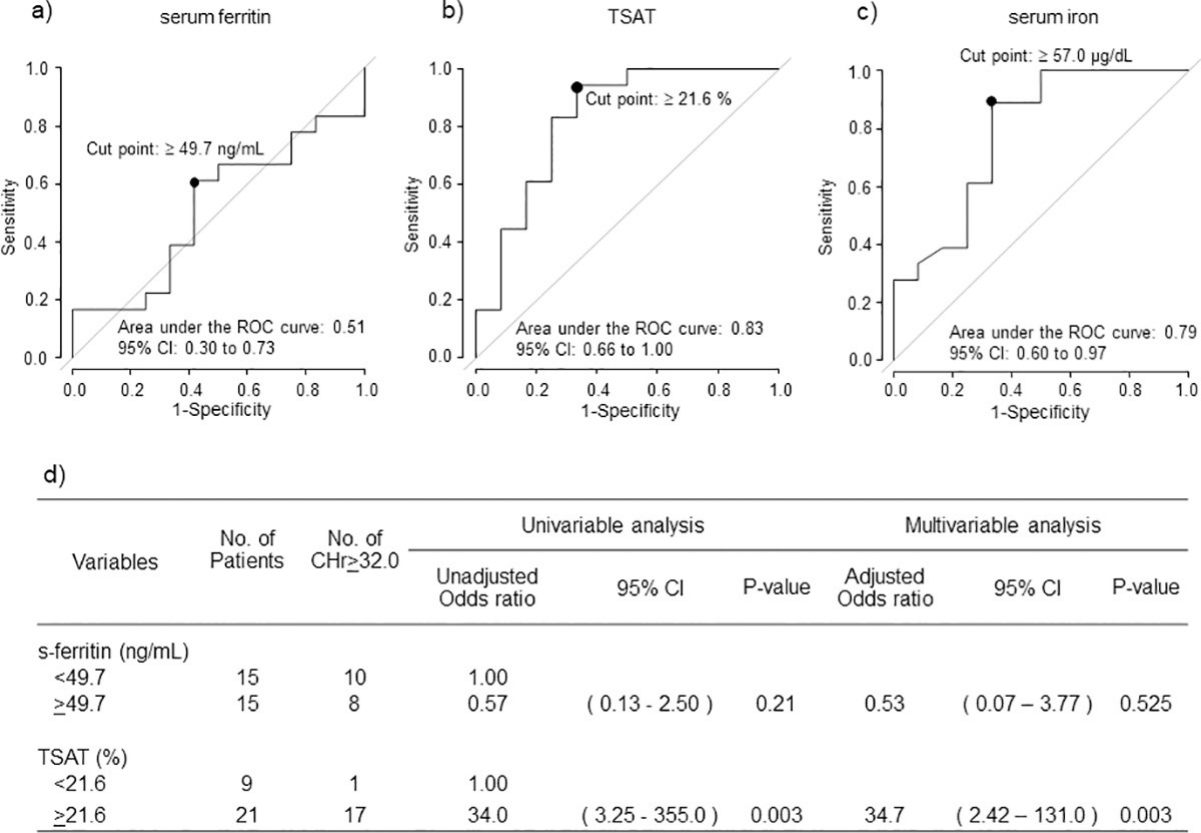
CHr levels and iron status on Day 0. ROC curves of (a) serum ferritin (b) TSAT and (c) s-Fe for CHr ≥ 32.0 pg on Day 0. The respective cutoff values for s-ft, TSAT, and s-Fe were ≥ 49.7 ng/mL (sensitivity 58.3%, specificity 61.1%, AUC 0.51), 21.6% (sensitivity 66.7%, specificity 94.4%, AUC 0.83, 95% CI 0.66–1.00), and 57.0 μg/dL (sensitivity 66.7%, specificity 94.4%, AUC 0.79, 95% CI 0.60–0.97). (d) Logistic model analysis with CHr ≥ 32.0 pg as the endpoint.

Both univariable and multivariable logistic model analyses with CHr ≥ 32.0 pg on Day 0 as the endpoint showed that the odds ratio was not significantly different for s-ft ≥ 49.7 ng/mL compared with s-ft < 49.7 ng/mL on Day 0. The odds ratio was significant higher for TSAT ≥ 21.6% compared with TSAT < 21.6% on Day 0 in both univariable analysis (34.0, 95% CI 3.25–355.0, p = 0.003) and multivariable analysis (34.7, 95% CI 2.42–131.0, p<0.003) (Fig 1).

### CHr levels and iron status on Day 28

According to ROC analysis with CHr ≥ 32.0 pg on Day 28 set as the endpoint, the cutoff value for s-ft was ≥ 35.5 ng/mL (sensitivity 68.4%, specificity 81.8%, AUC 0.74, 95% CI 0.55–0.93), whereas the cutoff values for TSAT and s-Fe were ≥ 16.2% (sensitivity 89.5%, specificity 81.8%, AUC 0.90, 95% CI 0.77–1.00) and 61.0 μg/dL (sensitivity 94.7%, specificity 72.7%, AUC 0.86, 95% CI 0.72–1.00) on Day 28.

**Fig 2:**
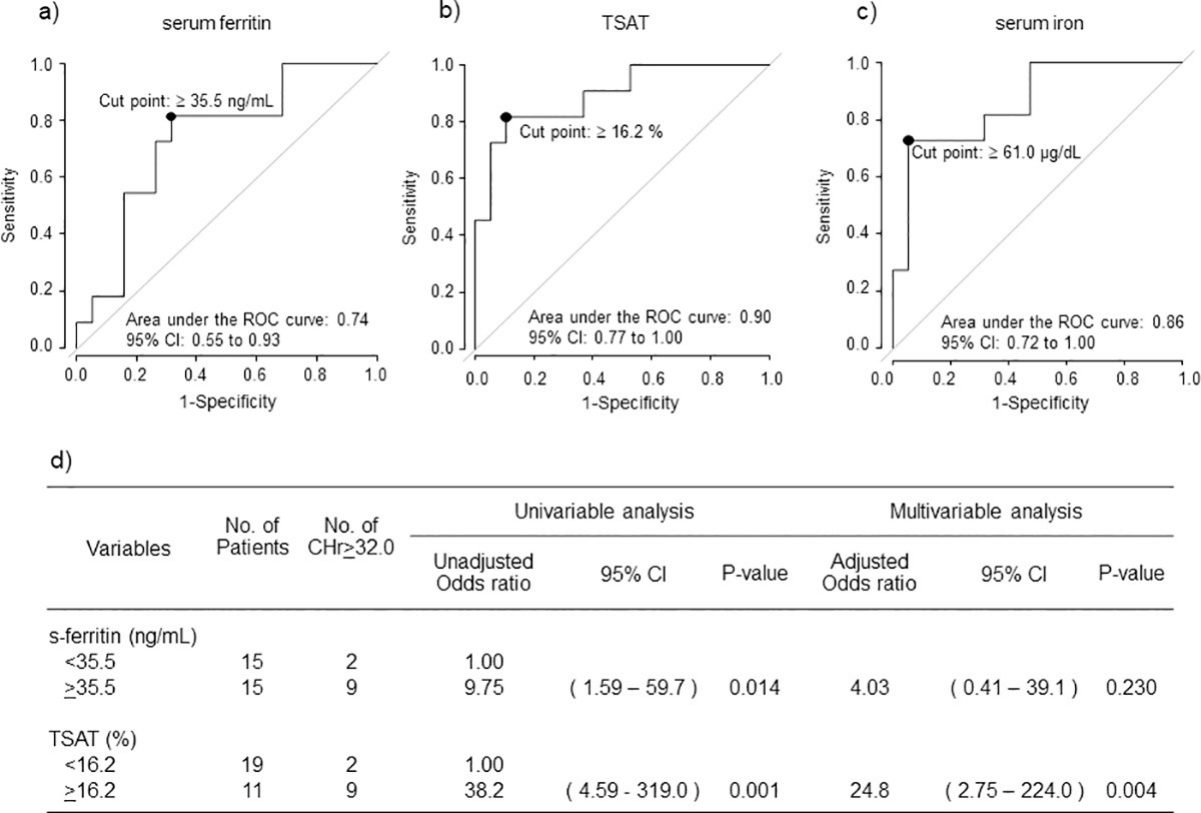
CHr levels and iron status on Day 28. ROC curves of (a) serum ferritin, (b) TSAT and (c) s-Fe for CHr ≥ 32.0 pg on Day 28. The respective cutoff values for s-ft and TSAT were ≥35.5 ng/mL (sensitivity 68.4%, specificity 81.8%, AUC 0.74, 95% CI 0.55–0.93), 16.2% (sensitivity 89.5%, specificity 81.8%, AUC 0.90, 95% CI 0.77–1.00) and 61.0 μg/dL (sensitivity 94.7%, specificity 72.7%, AUC 0.86, 95% CI 0.72–1.00). (d) Logistic model analysis with CHr ≥ 32.0 pg as the endpoint.

Logistic model analysis with CHr ≥ 32.0 pg set as the endpoint showed that the odds ratio for s-ft ≥ 35.5 ng/mL compared with s-ft < 35.5 ng/mL was significantly higher in univariable analysis (9.75, 95% CI 1.59–59.7, p = 0.014) but not in multivariable analysis. The odds ratio for TSAT ≥ 16.2% compared with TSAT < 16.2% was significantly higher in univariable analysis (38.2, 95% CI 4.59–319.0, p = 0.001) and in multivariable analysis (24.8, 95% CI 2.75–224.0, p = 0.004) (Fig 2).

### CHr levels on Day 28 and iron status on Day 0

According to ROC analysis with CHr ≥ 32.0 pg on Day 28 set as the endpoint, the cutoff value for s-ft on Day 0 was ≥ 81.6 ng/mL (sensitivity 84.2%, specificity 63.6%, AUC 0.68, 95% CI 0.45–0.92), whereas the cutoff value for TSAT and s-Fe on Day 0 were ≥ 23.9% (sensitivity 63.2%, specificity 81.8%, AUC 0.68, 95% CI 0.48–0.88) and 58.0 μg/dL (sensitivity 52.6%, specificity 90.9%, AUC 0.68, 95% CI 0.47–0.89).

**Fig 3:**
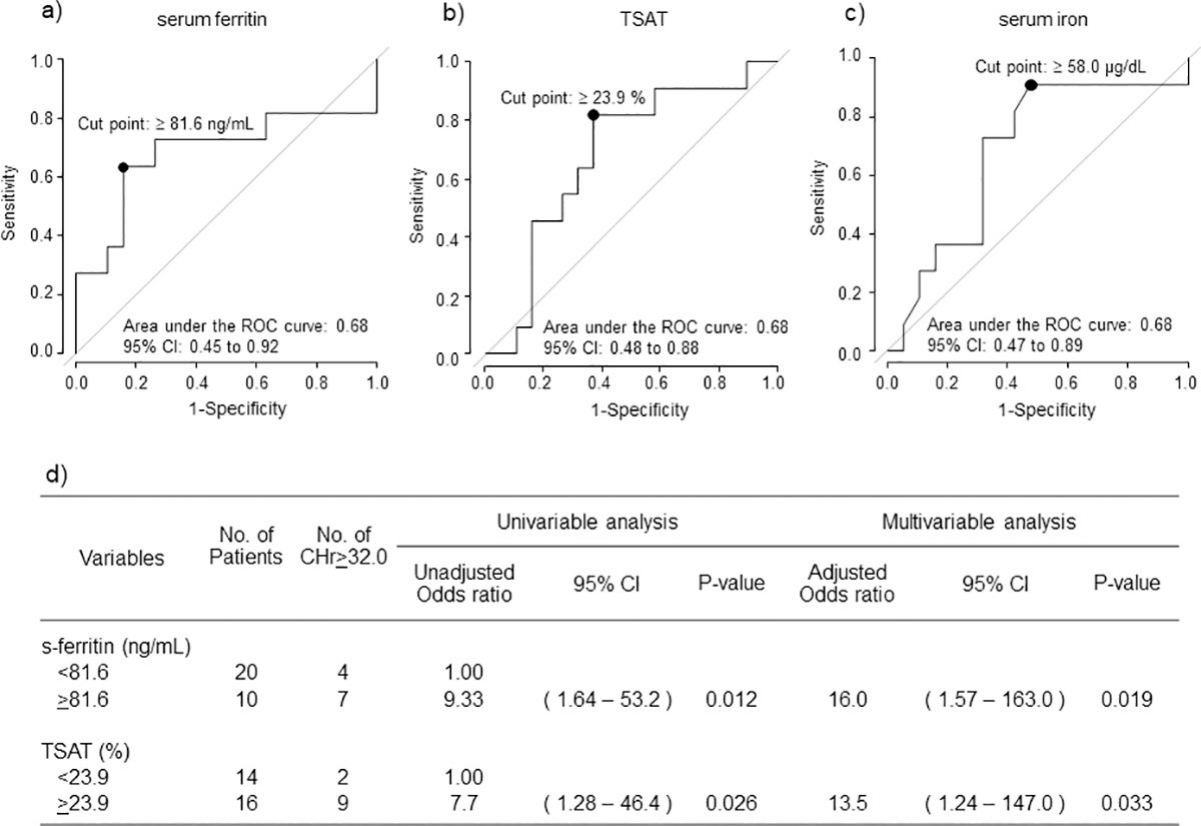
CHr levels on Day 28 and iron status on Day 0. ROC curves of (a) serum ferritin, (b) TSAT and (c) s-Fe on Day 0 for CHr ≥ 32.0 pg on Day 28. The respective cutoff values for s-ft, TSAT and s-Fe were at least at least 81.6 ng/mL (sensitivity 84.2%, specificity 63.6%, AUC 0.68, 95% CI 0.45–0.92), 23.9% (sensitivity 63.2%, specificity 81.8%, AUC 0.68, 95% CI 0.48–0.88) and 58.0 μg/dL (sensitivity 52.6%, specificity 90.9%, AUC 0.68, 95% CI 0.47–0.89), respectively. (d) Logistic model analysis with CHr ≥ 32.0 pg as the endpoint.

Logistic model analysis with CHr ≥ 32.0 pg on Day 28 set as the endpoint showed that the odds ratio for s-ft ≥ 81.6 ng/mL compared with s-ft < 81.6 ng/mL on Day 0 was significantly higher in univariable analysis (9.33, 95% CI 1.64–53.2, p = 0.012] and in multivariable analysis (16.0, 95% CI 1.57–163.0, p = 0.019). The odds ratio for TSAT ≥ 23.9% compared with TSAT < 23.9% on Day 0 was significantly higher in univariable analysis (7.7, 95% CI 1.28–46.4, p = 0.026) and in multivariable analysis (13.5, 95% CI 1.24–147.0, p<0.033) (Fig 3).

### Correlation between change in CHr level and change in reticulocyte count from Day 0 to Day 28 / s-ft on Day 0

**Fig 4:**
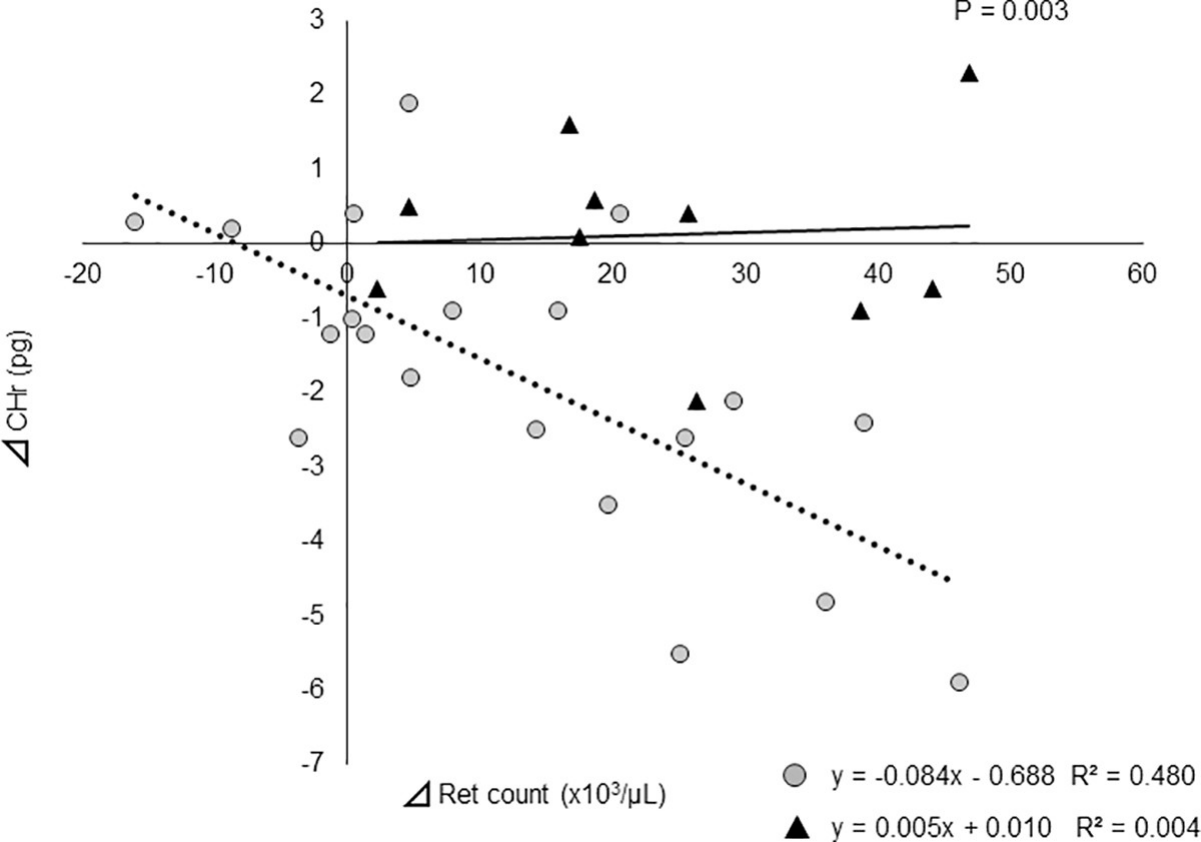
Correlation between the change in CHr level and change in reticulocyte count from Day 0 to Day 28. Change in CHr level showed a strong negative correlation with change in reticulocyte count in the low group (s-ft < 81.6 ng/mL) (p<0.001) but showed no correlation in the high group (s-ft ≥ 81.6 ng/mL) (p = 0.868). The slopes obtained by the linear regression analysis showed a significant difference between the two groups (p = 0.003).

Patients were divided into a low s-ft group (< 81.6 ng/mL) and high s-ft group (≥ 81.6 ng/mL). In the low s-ft group, there was a strong negative correlation between CHr level and Ret count (r = −0.084, R2 = 0.480, p<0.001). However, in the high s-ft group, there was almost no change in CHr level with increasing Ret coun. The correlation between change in CHr level and change in reticulocyte count from Day 0 to Day 28 was significantly different between the two groups based on the slopes obtained for the linear regression models (p = 0.003) (Fig 4).

### Factors related to change in Ret count from Day 0 to Day 28

**Fig 5:**
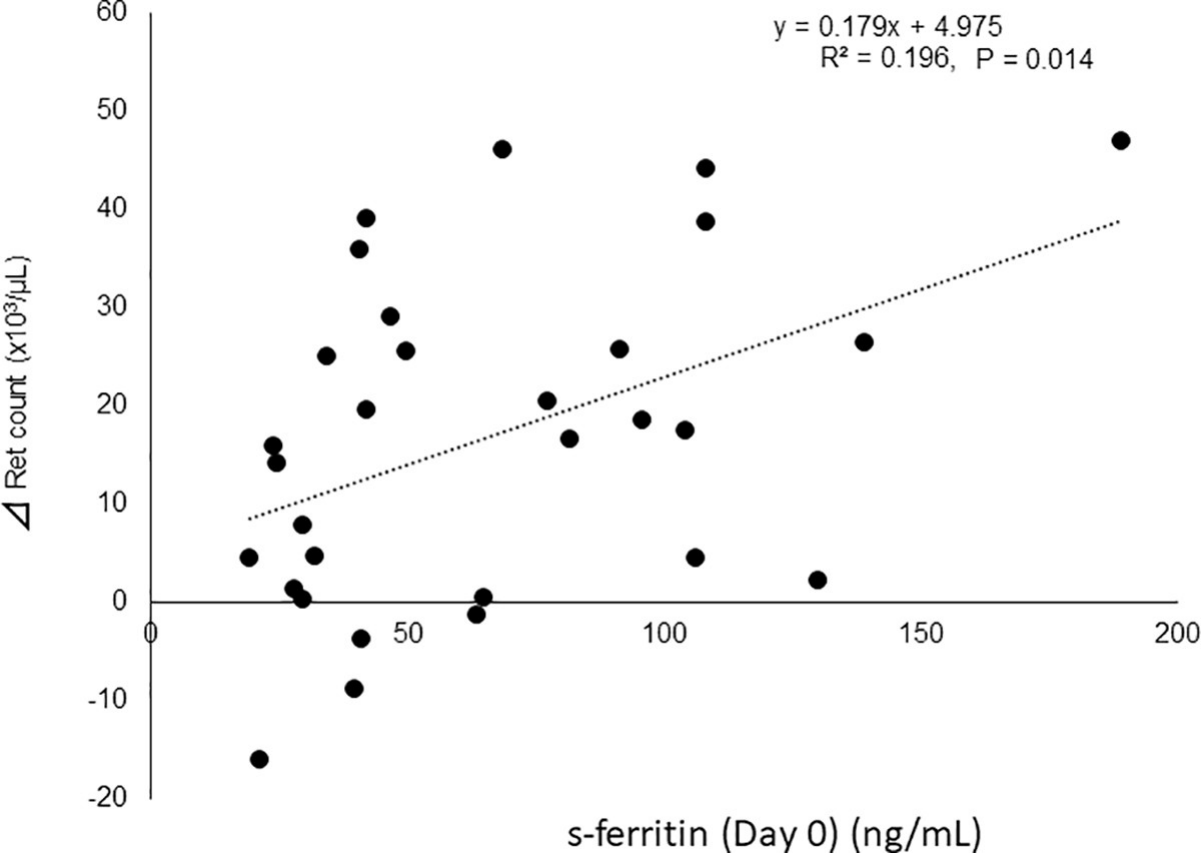
Correlation between change in Ret count from Day 0 to Day 28 and s-ft on Day 0. Change in Ret count showed a strong positive correlation with s-ft on Day 0 (p = 0.014).

The multiple linear regression model showed significantly positive correlation between change in Ret count and only s-ft on Day 0, with a β-coefficient of 0.18 (95% CI 0.04 to 0.32, p = 0.014) (Fig 5). The change in Ret count was not correlated with TSAT on Day 0 or the change in EPO concentration.

## Discussion

We previously examined hematopoiesis and iron metabolism during the 28-day period after switching from DA to Rox. Monitoring mature erythrocytes does not necessarily reflect real-time changes because they have a lifespan of 120 days, so a Ret marker was used for more detailed analysis of changes. We found increases in hematopoiesis and the rate of iron utilization immediately after the switch to Rox, indicating higher iron consumption when Rox was administered than when DA was administered [17]. As shown Table 2, the Hb level was significantly increased on Day 28, suggesting the effectiveness of Rox. On the other hand, the levels of MCV and CHr were significantly decreased, suggesting possible iron deficiency in this population with median s-ft level of 48.2 ng/mL and mean TSAT of 25.5%. Iron supplementation is essential for effective treatment of anemia, and it is therefore important to investigate the correlation between hematopoiesis and iron status. Ret cells are differentiated erythroblasts immediately after hemoglobulin synthesis. They stay within the bone marrow for 2–3 days and then enter the circulation where their maturation completes within 1–2 days. Thus, CHr level is considered to reflect the amount of iron recently used for hematopoiesis. Because CHr level is measured directly by flow cytometry based on the Ret count and Hb level, no influences other than those associated with hematopoiesis and iron metabolism are expected, and thus CHr level is thought to be more useful than s-ft level and TSAT for diagnosing iron deficiency [19, 20].

In this study, to avoid iron deficiency during Rox therapy, we examined the association of CHr level with iron status during DA therapy and Rox therapy. According to the results of a study investigating iron deficiency (CHr cutoff value 32 pg, sensitivity 100%, specificity 90%) based on hematocrit level in response to the intravenous iron injection [21], we defined CHr < 32.0 pg as iron deficiency and investigated the iron status associated with CHr ≥ 32.0 pg. Because the package insert of Rox recommends continuation of the initial oral dose for 4 weeks, data on Day 0 (reflecting the status during DA therapy) and Day 28 (reflecting the status during Rox therapy) were collected and analyzed.

We found that, on Day 0, the effect of s-ft level was negligible, and CHr ≥ 32.0 pg was likely to be maintained when TSAT was ≥ 21.6%. This suggests that when anemia is managed well by DA with favorable iron metabolism (TSAT ≥ 21.6%), a sufficient amount of iron for hematopoiesis is ensured even with a small iron stock. On the other hand, on Day 28 (during Rox therapy), the strong effect of TSAT remained, but a possible effect of s-ft level was found. Also, while the cutoff value for s-Fe was slightly higher on Day 28 than on Day 0, cutoff values for both s-ft and TSAT were lower on Day 28 than on Day 0. Suppression of hepcidin production and induction of ferroportin production by HIF are likely to facilitate the release of intracellular iron from cells, and inducing production of transferrin and transferrin receptors is likely to enhance iron transport and iron uptake by erythroblasts. This study also showed significant decreases in hepcidin level and increases in TIBC level, indicating that the low cutoff value for TSAT was due to increases in the TIBC level exceeding increases in the s-Fe level; thus, the amount of iron necessary for Hb synthesis was likely to be ensured even with low TSAT. Because marked increases in TIBC occurred during Rox therapy, the TSAT value corresponding to iron deficiency is predicted to be lower than the current value. The effect of s-ft level on CHr level can be explained by increases in iron demand in response to enhanced hematopoiesis after switching to Rox, and consequent dependence on iron stock as iron supply. This study suggested that Rox therapy might affect the conventional standard values of TSAT and s-ft.

We next examined the association of CHr level on Day 28 with s-ft level and TSAT on Day 0 to find the iron status at the time of the switch to Rox for avoiding iron deficiency on Day 28.

ROC curve analysis revealed an s-ft cutoff value of 81.6 ng/mL and a TSAT cutoff value of 23.9%, and logistic reanalysis using these cutoff values showed high odds ratio for the CHr ≥ 32.0 pg in the group with s-ft level or TSAT at or above the corresponding cutoff value than in the group with s-ft level or TSAT value below the corresponding cutoff value. These results suggest that, ideally, s-ft level and TSAT should be maintained at ≥81.6 ng/mL and ≥23.9%, respectively, when switching from DA to Rox in order to avoid iron deficiency during the first 4 weeks of Rox treatment.

CHr level decreases as Ret count increases if iron is deficient, but is not influenced by Ret count if the amount of iron is sufficient [22]. Thus, we divided the subject into a group with s-ft level below the cutoff value on Day 0 and a group with a s-ft level at or above the cutoff value on Day 0, and compared the relationship of change in CHr level from Day 0 to Day 28 with change in Ret count between the groups. A strong negative correlation was shown in the group with s-ft < 81.6 ng/mL, while no correlation was noted in the group with s-ft ≥ 81.6 ng/mL, indicating significant differences in kinetics between the groups. In other words, if s-ft level is ≥ 81.6 ng/mL at the time of switching to Rox, iron supply is likely to be sufficient for hematopoiesis even though the Ret count increases.

Further, we examined patient characteristics, TSAT on Day 0, Rox dose per body weight, and changes in EPO concentration to reveal whether these factors were associated with change in Ret count, and we found a significant positive correlation with only s-ft level on Day 0. This indicates the possibility that higher iron stock in the body more strongly potentiates the effect of Rox on hematopoiesis. A phase 3 study including Japanese patients on dialysis found that the upper end of the standard deviation of Hb level during weeks 2–8 of Rox therapy exceeded 12.0 g/dL [15], raising the concern of overshooting the target early in Rox therapy. Because of this, although iron deficiency is a concern during Rox therapy, especially in the introduction phase, it seems advisable to administer iron agents with caution.

This study showed elevated hematopoiesis and increased iron consumption after the switch to Rox. The iron requirement varies depending on hematopoietic status, and the amount of blood produced does not greatly change during the stable phase. Rox improved iron utilization efficiency, suggesting that favorable Hb synthesis can be maintained with a smaller amount of iron in the stable phase of Rox therapy compared with DA therapy.

This study is limited to the early stage after switching to Rox, so long-term optimal iron status during Rox therapy needs to be investigated in the future.

## Limitations

This was a single-center study with a small sample size, and the influence of controlling iron at low levels cannot be ruled out. The number of patients was limited because ESA is a well-established treatment for renal anemia and it is difficult to select another treatment outside of a clinical trial setting. Also, renal anemia is a relatively stable condition in patients managed under the same treatment policy at the same facility.

## Conclusion

This study showed a strong dependence of Hb synthesis on TSAT during both DA therapy and Rox therapy, although Hb synthesis was maintained with low TSAT and s-ft during Rox therapy compared with during DA therapy, suggesting that the iron status enabling effective hematopoiesis differs between DA therapy and Rox therapy. Rox therapy significantly decreases in hepcidin level and increases in TIBC level. Thus, it was suggested that TSAT and s-ft values during Rox therapy might differ from the conventional standard values. Iron utilization and hematopoiesis were increased immediately after the switch from DA to Rox, and thus the iron status with s-ft ≥ 81.6 ng/mL and TSAT ≥ 23.9% is desirable for avoiding iron deficiency during the first 4 weeks of Rox therapy.

## Supporting information

S1 Fig (TIF)

S2 Fig (TIF)

S3 Fig (TIF)

S4 Fig (TIF)

S5 Fig (TIF)
