# Supplementary figures and images for: Hypoxia-inducible factor prolyl hydroxylase domain inhibitor may maintain hemoglobin synthesis at lower serum ferritin and transferrin saturation levels than darbepoetin alfa
Source: PLoS One. 2021 Jun 18;16(6):e0252439. doi: 10.1371/journal.pone.0252439 (PMC8213169; doi:10.1371/journal.pone.0252439)

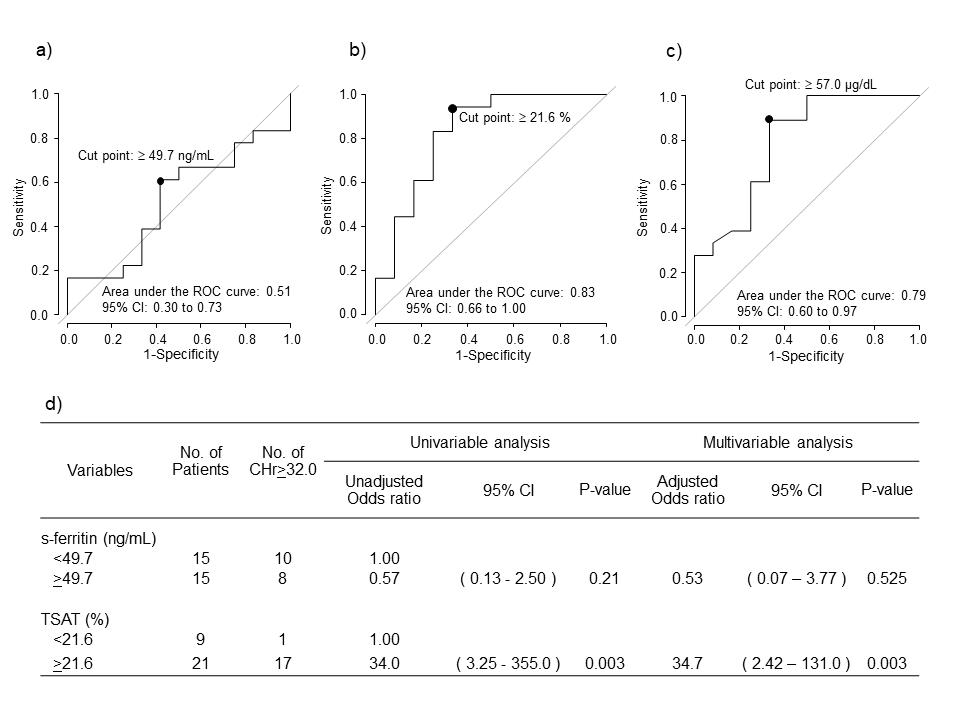

Supplement: S1 Fig — (TIF) [file pone.0252439.s001.tif]

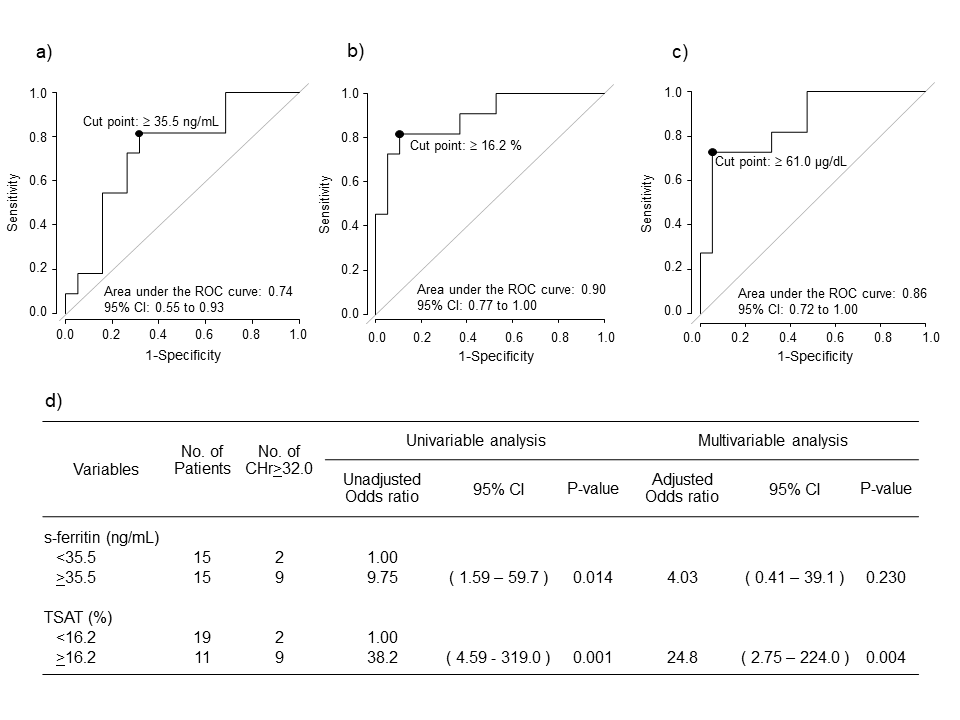

Supplement: S2 Fig — (TIF) [file pone.0252439.s002.tif]

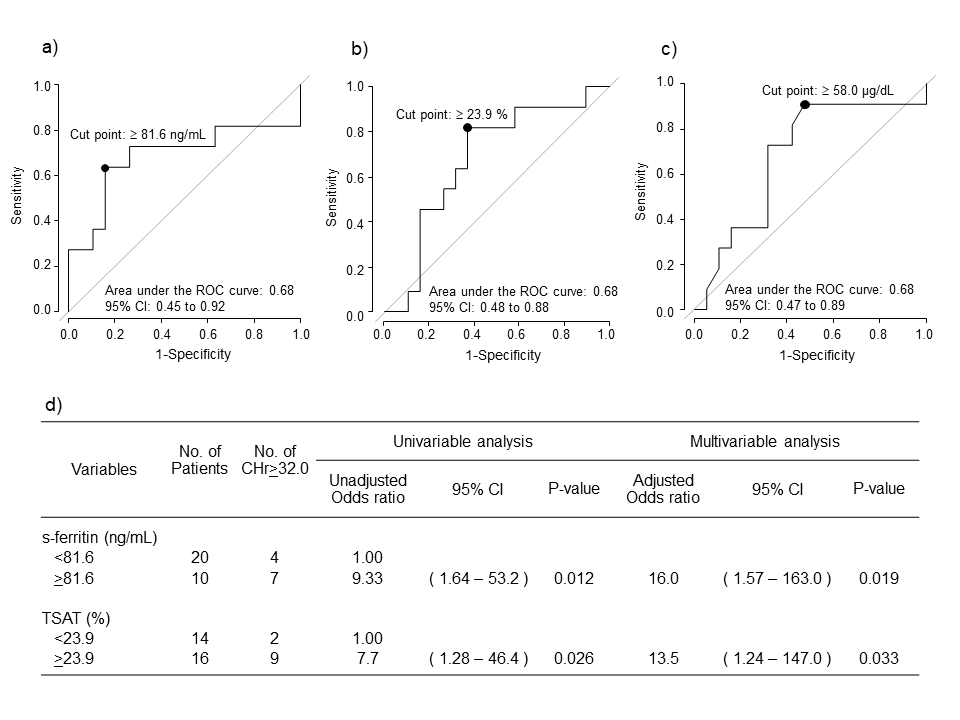

Supplement: S3 Fig — (TIF) [file pone.0252439.s003.tif]

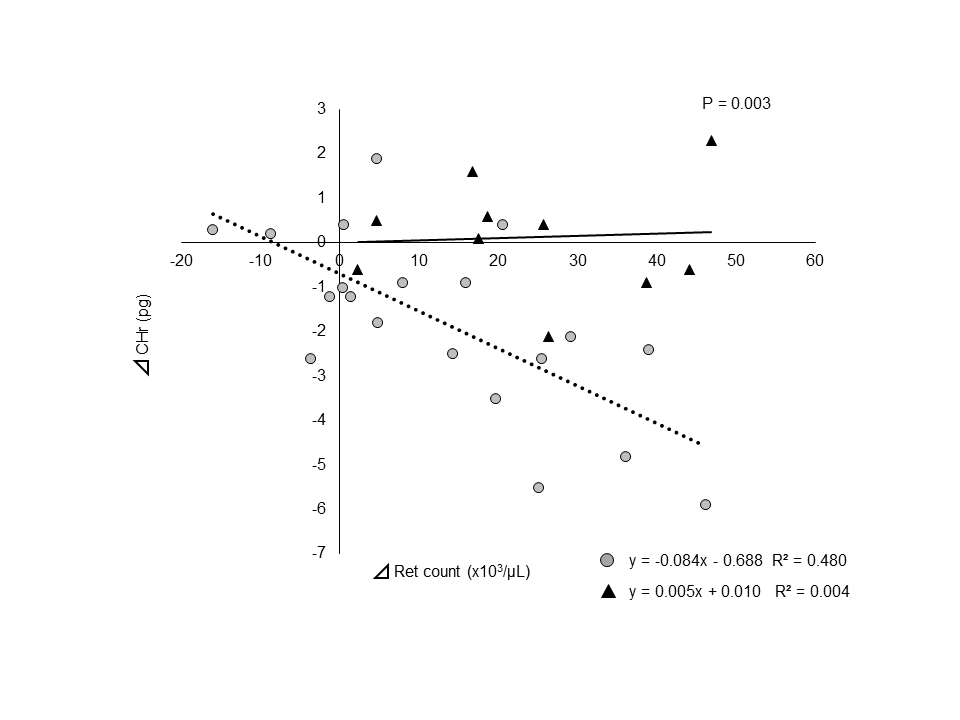

Supplement: S4 Fig — (TIF) [file pone.0252439.s004.tif]

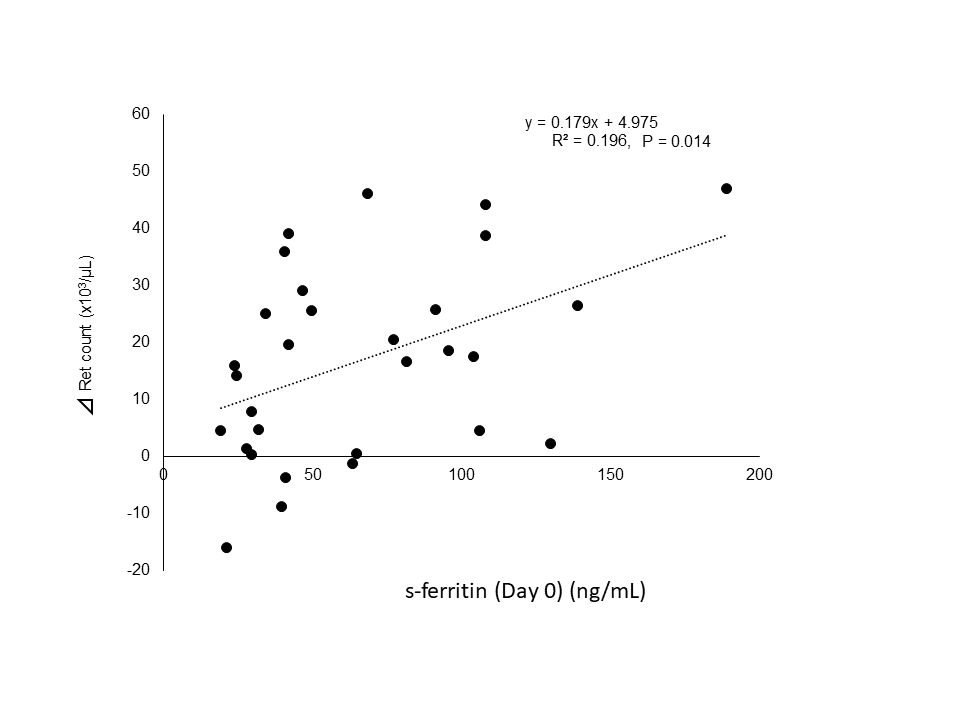

Supplement: S5 Fig — (TIF) [file pone.0252439.s005.tif]
